# Overexpression of klotho suppresses growth and pulmonary metastasis of osteosarcoma in *vivo*

**DOI:** 10.1590/1678-4685-GMB-2019-0229

**Published:** 2020-04-29

**Authors:** Ying Li, Hai-jun Xiao, Feng Xue

**Affiliations:** 1 Shanghai Fengxian District Central Hospital, Department of Orthopedics, Shanghai, China

**Keywords:** Osteosarcoma, klotho, growth, metastasis

## Abstract

Klotho is originally discovered as an anti-aging gene and knock-out of klotho accelerates aging in mice. Subsequent studies support the anti-carcinogenesis role of klotho in a variety of human malignancies. The present study investigated the role of klotho on growth and metastasis of osteosarcoma cells. The osteosarcoma cells were transduced with lentivirus particles encoding klotho or scramble control. The reconstructed osteosarcoma cells were injected into the femoral medullary cavity of nude mice to establish a xenograft animal model. The anti-tumor properties of klotho were evaluated in terms of tumor growth, apoptosis, glycogen production, and pulmonary metastasis. Lentivirus-mediated overexpression of klotho significantly decreased tumor volume and weight in osteosarcoma mice. Determination of PCNA and Ki67 expression revealed that overexpression of klotho inhibited cell proliferation in tumor tissues obtained from osteosarcoma xenografts. PAS staining also showed that overexpression of klotho significantly decreased the production of glycogen in osteosarcoma. Moreover, TUNEL positive cells were significantly increased after lentivirus-mediated overexpression of klotho. Furthermore, lentivirus-mediated upregulation of klotho reduced the number of pulmonary metastatic lesions in mice compared to control mice. These findings demonstrated that elevated klotho could inhibit osteosarcoma cell growth and pulmonary metastasis *in vivo*, suggesting that klotho may be a valuable therapeutic target for osteosarcoma.

## Introduction

Osteosarcoma is the most common primary bone malignancies, accounting for 60% of all bone sarcoma. Osteosarcoma has a worldwide incidence of approximately 1∼3 annually per million with 50% of 5-year overall survival. However, the survival of patients with metastatic osteosarcoma has remained only 20% with an overall 5-year survival rate (Kager *et al.*, 2017; Harrison *et al.*, 2018). Current treatment strategy includes surgical resection and chemotherapy. Systematic toxicity induced by chemotherapy often leads to patient's health deterioration. Thus, new therapeutic targets with specific targeting cancer cells are urgently needed to be developed.

Klotho is a membrane-bound protein that primarily expressed in the distal tubule of the kidney (Ide *et al.*, 2016). Moreover, klotho is also detected in the proximal tubule, choroid plexus, parathyroid gland, and sinoatrial node. The extracellular part of Klotho contains two homologous domains that share sequences with a high similarity (Takeshita *et al.*, 2004). Studies in animal models suggest that abnormal expression of klotho leads to a syndrome that resembles human aging, including a short lifespan, osteoporosis, arteriosclerosis, infertility, emphysema, and skin atrophy (Kuro-o *et al.*, 1997). In addition, klotho expression is dysregulated in several human malignancies (Zeldich *et al.*, 2015). Further studies have shown that overexpression of klotho plays a suppressive role in tumorigenesis and progression in ovarian, breast, cervical, pancreatic, colorectal and lung cancers (Zhou and Wang, 2015; Aviel-Ronen *et al.*, 2016; Li *et al.*, 2018). However, the precise role of klotho is osteosarcoma has not been evaluated. Therefore, the present study, for the first time, explored the effects of klotho in osteosarcoma proliferation and metastasis.

## Material and Methods

### Cell culture

MNNG/HOS cells were obtained from the Shanghai Institute of Biological Sciences, Chinese Academy of Sciences (CAS, Shanghai, China). Cells were maintained in Dulbecco's modified Eagle medium (DMEM) (Gibco, NY, USA) containing 10% fetal bovine serum, 100 μg/ml streptomycin, and 100 μg/ml penicillin (Hyclone, USA). Cells were cultured at 37 °C in a 5% CO_2_ humidity-controlled incubator.

### Xenograft animal model

All animal procedures were approved by the Animal Ethics Committee of our hospital. Six-week-old, SPF-grade nude mice were purchased from Shanghai Slak. Prior to injection, the mice were anesthetized with intraperitoneal injection of pentobarbital. Human osteosarcoma MNNG/HOS cells infected with lentivirus particles encoding klotho (Genechem, Shanghai, China) were injected into the femoral medullary cavity of nude mice. In addition, mice in the control group were injected with MNNG/HOS cells and no operative measures were taken on those mice in the blank group. Tumor dimensions (length, L and width, W) were measured and tumor volumes were then calculated as TV= (LW^2^)/2. Mice were euthanized followed by cervical dislocation at the end point and the tumor samples were measured and collected for subsequent experiments.

### PAS staining

Tissue samples were fixed in 4% paraformaldehyde, permeabilized with Tween 20, and then treated with amylase for 20 min. Cells were then treated with 1% periodic acid for 10 min and washed with running tap water to remove excess acid. Subsequently, cells were treated with Schiff's reagent for 20 min, followed by washing and counter staining with DAPI to stain the nucleus.

### Immunohistochemistry

The paraffin-embedded blocks were cut into 3-μm thickness sections and mounted on glass slides coated with poly-L-lysine, deparaffinized with xylene and rehydrated with gradient ethanol. The slides were heated and incubated in a 10 mM citrate buffer and incubated with antibodies against Ki-67 and PCNA. The immunostaining intensity was independently evaluated by two pathologists.

### *In vivo* pulmonary metastasis assay

Mice were killed and tumor samples were frozen in liquid nitrogen and stored at −80 °C for further analysis. Following fixation in neutral buffered formalin, paraffin-embedded specimens were cut 4μm thick and stained with hematoxylin and eosin.

### Apoptosis assays

Tumor samples were fixed in 4% paraformaldehyde, deparaffinized, washed with xylene and ethanol and fixed in 4% paraformaldehyde for 15 min. Each section was incubated with 20 μg/mL proteinase K solution for 10 min, washed and re-fixed in 4% paraformaldehyde for 5 min. TUNEL staining was performed using the Cell Death Detection Kit (Roche) according to the manufacturer's protocol. Three independent experiments were conducted

### Statistical analysis

Experimental data were analyzed using the GraphPad Prism program (GraphPad Software, Inc., San Diego, CA). Data are presented as mean ± S.E.M. Statistical significance was performed on the data using ANOVA. A value of P<0.05 was considered to be statistically significant.

## Results

### Overexpression of klotho inhibits osteosarcoma cell growth *in vivo*

**Figure 1:**
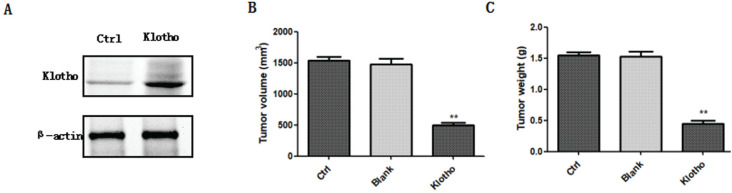
Overexpression of klotho inhibits osteosarcoma growth. Klotho overexpression in transduced osteosarcoma cells was confirmed by western blot (A). A final concentration of 10^7^ cells/ml of osteosarcoma cells with or without overexpression of klotho were injected into mice. Four weeks after injection, the tumor volume (B) and weight (C) were evaluated in each group. ** P<0.01, n=5 mice in each group.

**Figure 2:**
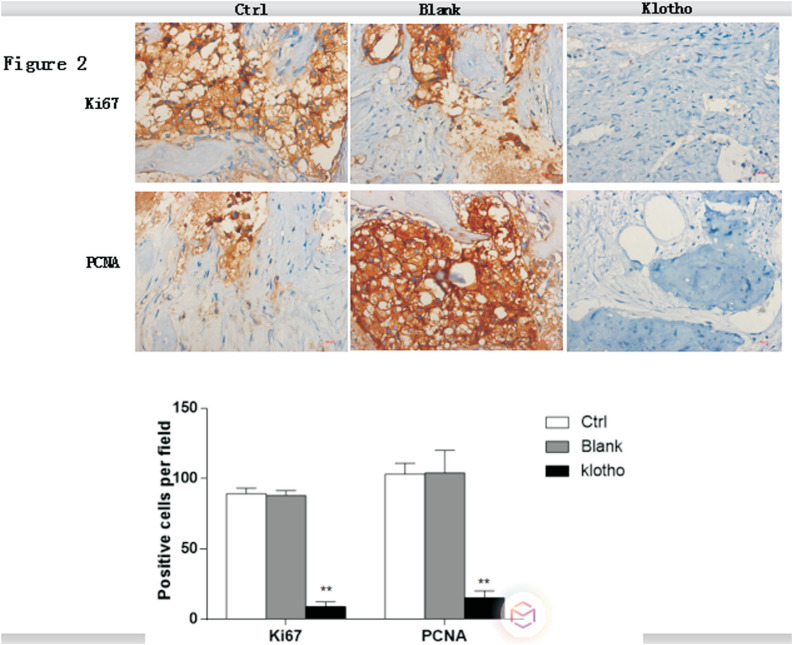
Overexpression of klotho inhibits osteosarcoma cell proliferation *in vivo*. A final concentration of 10^7^ cells/ml of osteosarcoma cells with or without overexpression of klotho were injected into mice. Four weeks after injection, immunohistochemistry was performed to detect the expression of PCNA and Ki67 in each group. ** P<0.01, n=5 mice in each group.

In order to determine whether klotho was involved in tumor growth, mice were injected with osteosarcoma cells with or without overexpression of klotho, and tumor volume and weight were evaluated. Klotho overexpression in transduced osteosarcoma cells was confirmed by western blot (Figure 1A). Four weeks after injection, we found that, compared to the control group, the tumor volume and weight in the blank group showed no significant changes (P>0.05). However, lentivirus-mediated overexpression of klotho significantly decreased tumor volume and weight in the mice model of osteosarcoma (Figure 1B and C). Furthermore, we examined the effects of klotho on cell proliferation in tumor tissues derived from control and klotho overexpressed mice using anti-PCNA or anti-Ki67 antibody (Figure 2). PCNA and Ki67 are the markers of cell proliferation. Overexpression of klotho inhibited cell proliferation in tumor tissues obtained from xenografts compared to control mice, as measured by immunohistochemistry (IHC). Collectively, these data suggested the anti-proliferative role of klotho in osteosarcoma.

### Overexpression of klotho inhibits glycogen production in osteosarcoma

**Figure 3:**
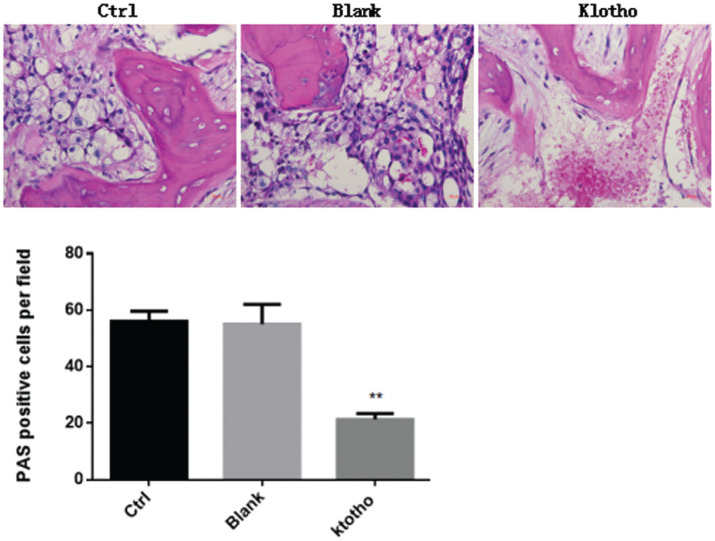
Overexpression of klotho inhibits glycogen production in osteosarcoma. A final concentration of 10^7^ cells/ml of osteosarcoma cells with or without overexpression of klotho were injected into mice. Four weeks after injection, PAS staining was used to assess the glycogen content of osteosarcoma. ** P<0.01, n=5 mice in each group.

To determine whether klotho was associated with glycogen production in tumor cells, mice were injected with osteosarcoma cells with or without overexpression of klotho. Then, PAS staining was used to assess the glycogen content of osteosarcoma with or without overexpressed klotho. We observed no significant changes in glycogen storage between control and blank groups (P>0.05). However, overexpression of klotho significantly decreased the PAS-positive cells per field and thus suppressed the production of glycogen in osteosarcoma (Figure 3).

### Overexpression of klotho induces apoptosis in osteosarcoma

**Figure 4:**
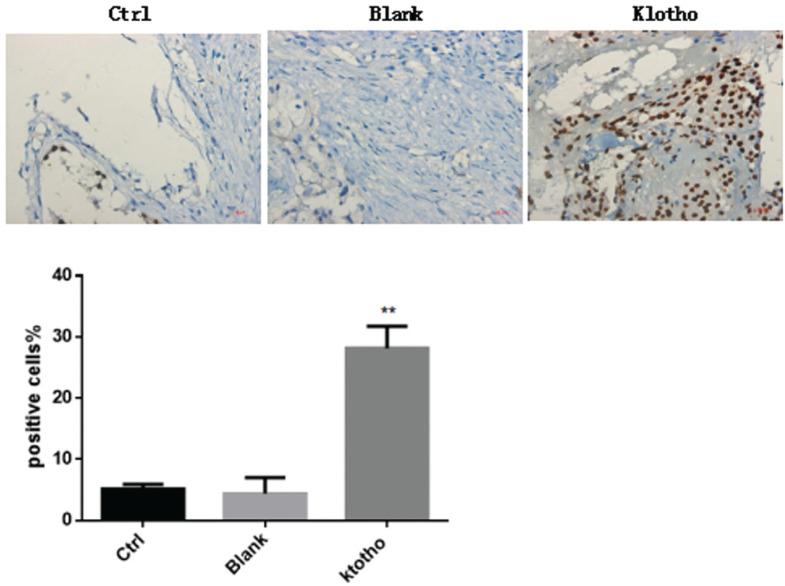
Overexpression of klotho induces apoptosis in osteosarcoma. A final concentration of 10^7^ cells/ml of osteosarcoma cells with or without overexpression of klotho were injected into mice. Four weeks after injection, TUNEL staining was used to assess the apoptotic cell death in each group. ** P<0.01, n=5 mice in each group.

To determine whether up-regulation of klotho was associated with the induction of apoptosis, mice were injected with osteosarcoma cells with or without overexpression of klotho. Then, TUNEL staining was used to assess the apoptotic cell death induced by klotho. Consequently, we detected no significant changes in TUNEL positive cells between control and blank groups (P>0.05). Nevertheless, lentivirus-mediated overexpression of klotho obviously suppressed the TUNEL-positive cell number, suggesting that enhanced expression of klotho was associated with induction of apoptosis in osteosarcoma (Figure 4).

### Overexpression of klotho inhibits lung metastasis of osteosarcoma *in vivo*

**Figure 5:**
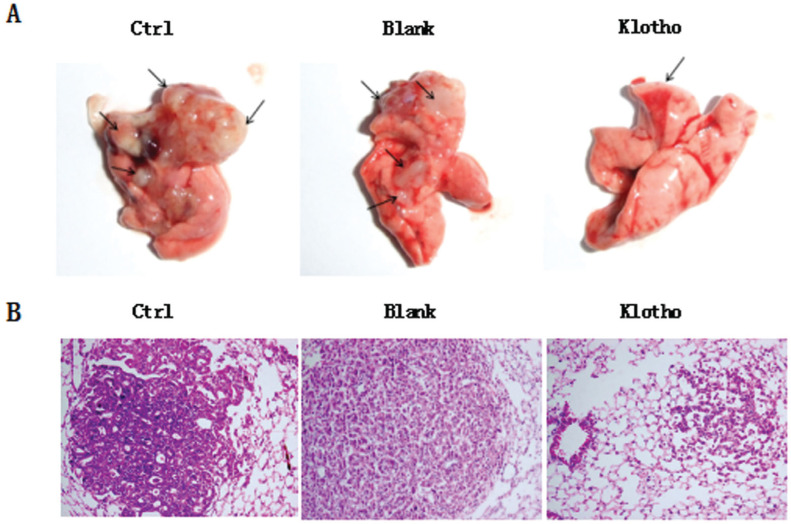
Overexpression of klotho inhibits lung metastasis of osteosarcoma *in vivo*. A final concentration of 10^7^ cells/ml of osteosarcoma cells with or without overexpression of klotho were injected into mice. Four weeks after injection, the general morphology and tumor number were observed (arrow indicated tumors) (A). And metastatic volume of tumors was analyzed by HE staining in each group (B). ** P<0.01, n=5 mice in each group.

To clarify the role of klotho in osteosarcoma metastasis *in vivo*, mice were injected with osteosarcoma cells with or without overexpression of klotho. Subsequently, all injected tumor cells formed large primary tumors at the injection site. Examination of the lungs revealed a high number and volume of metastatic lesions in mice injected with control and blank cells. However, there was a marked reduction in metastases produced by cells overexpressing klotho (Figure 5A and B). These data suggested that enforced expression of klotho could suppress the spontaneous pulmonary metastasis of osteosarcoma *in vivo*.

## Discussion

Klotho is firstly discovered as an anti-aging gene and knock-out of *klotho* accelerates aging in mice (Kurosu *et al.*, 2005). Subsequently, accumulating evidence support that klotho has a wide range of biological effects, including anti-aging, anti-apoptosis, calcium and phosphorus metabolism, anti-carcinogenesis, and anti-oxidation (Abraham *et al.*, 2016; Boksha *et al.*, 2017). In our study, overexpression of klotho exhibited a significant anti-oncogenic role in osteosarcoma.

Firstly, we investigated whether klotho is associated with tumor growth of osteosarcoma. Previous studies suggest that klotho inhibits many pathways involved in carcinogenesis, including PI3K/Akt/mTOR signaling, Wnt/β-catenin signaling, ERK1/2 signaling, and Smad2/3 signaling (Tang *et al.*, 2016; Lim *et al.*, 2017). It is reported that the anti-tumor effects of klotho are found to include the inhibition of proliferation. It is shown that klotho can suppress tumor growth and improve survival in several human malignancies, including lung, pancreatic, breast and colorectal carcinoma (Chen *et al.*, 2016; Mencke *et al.*, 2017; Yan *et al.*, 2017). Notably, *klotho*-deficient mice present several bone-associated problems. It has been reported that klotho deficiency *in vivo* leads to overactivation of Wnt signaling, resulting in stem cell senescence and a complex bone phenotype, which could be prevented by klotho overexpression in this model (Liu *et al.*, 2007). Conversely, klotho preservation attenuates chronic kidney disease-associated bone injury in mice via inhibition of histone deacetylase (Lin *et al.*, 2017). In addition, klotho is a co-receptor of FGF23, an endocrine FGF expressed and secreted by osteocytes, increases the affinity for FGF23 and potentiating downstream signaling that potentiates phosphaturia and inhibits activation of vitamin D (Kurosu *et al.*, 2006; Urakawa *et al.*, 2006; Guan *et al.*, 2014). However, the role of klotho in the pathogenesis of osteosarcoma has never been reported. In our study, we found that lentivirus-mediated overexpression of klotho significantly decreased tumor volume and weight in osteosarcoma mice. Molecularly, IHC analysis of PCNA and Ki67 expression revealed that overexpression of klotho inhibited cell proliferation in tumor tissues obtained from osteosarcoma xenografts, supporting the anti-proliferative role of klotho in osteosarcoma. Excessive glycogen production is detected in tumor cells with altered metabolism. Thus, evaluation of glycogen production could reflect the status of cellular metabolism in tumors. PAS staining also showed that overexpression of klotho significantly decreased the production of glycogen in osteosarcoma. Evidence also shows that the anti-tumor effects of klotho are involved in induction of apoptosis (Xie *et al.*, 2013; Sun *et al.*, 2015; Dai *et al.*, 2016). Consistently, TUNEL positive cells were significantly increased after lentivirus-mediated overexpression of klotho. These findings demonstrated that enhanced expression of klotho was associated with suppression of growth and induction of apoptosis in osteosarcoma.

As reported previously, the anti-tumor effects of klotho are attributed to its capacity to suppress migration (Mencke *et al.*, 2017). For example, klotho inhibits transforming growth factor-β1 signaling and suppresses renal cancer metastasis in mice (Doi *et al.*, 2011). Another study suggests that klotho inhibits the capacity of cell migration and invasion in cervical cancer (Chang *et al.*, 2012). In addition, loss of *Klotho* leads to increased Wnt5A expression, filamin cleavage, and cell migratory potential during melanoma progression (Camilli *et al.*, 2011). Therefore, we evaluated the role of klotho in osteosarcoma metastasis *in vivo*. Subsequently, examination of the lungs revealed a low number of metastatic lesions in mice injected with lentivirus particles encoding klotho, compared to control and blank cells, supporting the fact that klotho overexpression could suppress the spontaneous pulmonary metastasis of osteosarcoma *in vivo*.

In conclusion, we have shown that elevated expression of klotho is able to suppress growth and pulmonary metastasis of osteosarcoma *in vivo*. These findings demonstrate that klotho may serve as a valuable therapeutic target for treatment of osteosarcoma.

## References

[B1] Aviel-Ronen S, Rubinek T, Zadok O, Vituri A, Avivi C, Wolf I, Barshack I (2016). Klotho expression in cervical cancer: differential expression in adenocarcinoma and squamous cell carcinoma. J Clin Pathol.

[B2] Abraham CR, Mullen PC, Tucker-Zhou T, Chen CD, Zeldich E (2016). Klotho is a neuroprotective and cognition-enhancing protein. Vitam Horm.

[B3] Boksha IS, Prokhorova TA, Savushkina OK, Tereshkina EB (2017). Klotho protein: its role in aging and central nervous system pathology. Biochemistry (Mosc).

[B4] Chen T, Ren H, Thakur A, Yang T, Li Y, Zhang S, Wang T, Chen M (2016). Decreased level of Klotho contributes to drug resistance in lung cancer cells: Involving in Klotho-Mediated Cell Autophagy. DNA Cell Biol.

[B5] Chang B, Kim J, Jeong D, Jeong Y, Jeon S, Jung SI, Yang Y, Kim KI, Lim JS, Kim C (2012). Klotho inhibits the capacity of cell migration and invasion in cervical cancer. Oncol Rep.

[B6] Camilli TC, Xu M, O'Connell MP, Chien B, Frank BP, Subaran S, Indig FE, Morin PJ, Hewitt SM, Weeraratna AT (2011). Loss of Klotho during melanoma progression leads to increased filamin cleavage, increased Wnt5A expression, and enhanced melanoma cell motility. Pigment Cell Melanoma Res.

[B7] Dai D, Wang Q, Li X, Liu J, Ma X, Xu W (2016). Klotho inhibits human follicular thyroid cancer cell growth and promotes apoptosis through regulation of the expression of stanniocalcin-1. Oncol Rep.

[B8] Doi S, Zou Y, Togao O, Pastor JV, John GB, Wang L, Shiizaki K, Gotschall R, Schiavi S, Yorioka N (2011). Klotho inhibits transforming growth factor-beta1 (TGF-beta1) signaling and suppresses renal fibrosis and cancer metastasis in mice. J Biol Chem.

[B9] Guan X, Nie L, He T, Yang K, Xiao T, Wang S, Huang Y, Zhang J, Wang J, Sharma K (2014). Klotho suppresses renal tubulo-interstitial fibrosis by controlling basic fibroblast growth factor-2 signalling. J Pathol.

[B10] Harrison DJ, Geller DS, Gill JD, Lewis VO, Gorlick R (2018). Current and future therapeutic approaches for osteosarcoma. Expert Rev Anticancer Ther.

[B11] Ide N, Olauson H, Sato T, Densmore MJ, Wang H, Hanai JI, Larsson TE, Lanske B (2016). In vivo evidence for a limited role of proximal tubular Klotho in renal phosphate handling. Kidney Int.

[B12] Kager L, Tamamyan G, Bielack S (2017). Novel insights and therapeutic interventions for pediatric osteosarcoma. Future Oncol.

[B13] Kurosu H, Yamamoto M, Clark JD, Pastor JV, Nandi A, Gurnani P, McGuinness OP, Chikuda H, Yamaguchi M, Kawaguchi H (2005). Suppression of aging in mice by the hormone Klotho. Science.

[B14] Kuro-o M, Matsumura Y, Aizawa H, Kawaguchi H, Suga T, Utsugi T, Ohyama Y, Kurabayashi M, Kaname T, Kume E (1997). Mutation of the mouse klotho gene leads to a syndrome resembling ageing. Nature.

[B15] Kurosu H, Ogawa Y, Miyoshi M, Yamamoto M, Nandi A, Rosenblatt KP, Baum MG, Schiavi S, Hu MC, Moe OW (2006). Regulation of fibroblast growth factor-23 signaling by klotho. J Biol Chem.

[B16] Liu H, Fergusson MM, Castilho RM, Liu J, Cao L, Chen J, Malide D, Rovira II, Schimel D, Kuo CJ (2007). Augmented Wnt signaling in a mammalian model of accelerated aging. Science.

[B17] Lin W, Li Y, Chen F, Yin S, Liu Z, Cao W (2017). Klotho preservation via histone deacetylase inhibition attenuates chronic kidney disease-associated bone injury in mice. Sci Rep.

[B18] Li Q, Li Y, Liang L, Li J, Luo D, Liu Q, Cai S, Li X (2018). Klotho negatively regulated aerobic glycolysis in colorectal cancer via ERK/HIF1alpha axis. Cell Commun Signal.

[B19] Lim SW, Jin L, Luo K, Jin J, Shin YJ, Hong SY, Yang CW (2017). Klotho enhances FoxO3-mediated manganese superoxide dismutase expression by negatively regulating PI3K/AKT pathway during tacrolimus-induced oxidative stress. Cell Death Dis.

[B20] Mencke R, Olauson H, Hillebrands JL (2017). Effects of Klotho on fibrosis and cancer: A renal focus on mechanisms and therapeutic strategies. Adv Drug Deliv Rev.

[B21] Sun H, Gao Y, Lu K, Zhao GM, Li XH, Li Zhu, Chang H (2015). Overexpression of Klotho suppresses liver cancer progression and induces cell apoptosis by negatively regulating wnt/beta-catenin signaling pathway. World J Surg Oncol.

[B22] Takeshita K, Fujimori T, Kurotaki Y, Honjo H, Tsujikawa H, Yasui K, Lee JK, Kamiya K, Kitaichi K, Yamamoto K (2004). Sinoatrial node dysfunction and early unexpected death of mice with a defect of klotho gene expression. Circulation.

[B23] Tang X, Wang Y, Fan Z, Ji G, Wang M, Lin J, Huang S, Meltzer SJ (2016). Klotho: a tumor suppressor and modulator of the Wnt/beta-catenin pathway in human hepatocellular carcinoma. Lab Invest.

[B24] Urakawa I, Yamazaki Y, Shimada T, Iijima K, Hasegawa H, Okawa K, Fujita T, Fukumoto S, Yamashita T (2006). Klotho converts canonical FGF receptor into a specific receptor for FGF23. Nature.

[B25] Xie B, Zhou J, Shu G, Liu DC, Zhou J, Chen J, Yuan L (2013). Restoration of klotho gene expression induces apoptosis and autophagy in gastric cancer cells: tumor suppressive role of klotho in gastric cancer. Cancer Cell Int.

[B26] Yan Y, Wang Y, Xiong Y, Lin X, Zhou P, Chen Z (2017). Reduced Klotho expression contributes to poor survival rates in human patients with ovarian cancer, and overexpression of Klotho inhibits the progression of ovarian cancer partly via the inhibition of systemic inflammation in nude mice. Mol Med Rep.

[B27] Zeldich E, Chen CD, Avila R, Medicetty S, Abraham CR (2015). The anti-aging protein Klotho enhances remyelination following cuprizone-induced demyelination. J Mol Neurosci.

[B28] Zhou X, Wang X (2015). Klotho: a novel biomarker for cancer. J Cancer Res Clin Oncol.

